# Co‐Designing an Easy‐Read Adult Social Care Outcomes Measure for Older People: Approach to and Reflections on Involving People Living With Dementia and Their Supporters

**DOI:** 10.1111/hex.70712

**Published:** 2026-06-10

**Authors:** Rasa Mikelyte, Keith Oliver, Rosemary Oliver, James Caiels, Stacey Rand, Elizabeth Field, Lucy Webster, Ann‐Marie Towers

**Affiliations:** ^1^ Centre for Health Services Studies University of Kent Canterbury UK; ^2^ Member of the public taking part in the co‐design working group Canterbury UK; ^3^ Care and Outcomes Research Centre University of Kent Canterbury UK; ^4^ Kent and Medway NHS and Social Care Partnership Trust Canterbury UK; ^5^ Current Affiliation: Cordis Bright London UK; ^6^ Current Affiliation: Health and Social Care Workforce Research Unit, The Policy Institute King's College London London UK

**Keywords:** assessment tools, co‐design, dementia, quality of life, Social care

## Abstract

**Background:**

In the UK, over half a million older people rely on publicly funded social care services to support their daily living needs. It is crucial to measure the quality of these services to ensure they meet the needs of those they support. The Adult Social Care Outcomes Toolkit (ASCOT) was developed to assess social care‐related quality of life (SCRQoL; Netten et al., 2012). However, many older individuals, particularly those living with dementia, face difficulties completing standard questionnaires (Aznar et al., 2021).

**Aim:**

This project aimed to enhance the accessibility of the ASCOT toolkit for older people, enabling more people to self‐report their experiences of social care.

**Methods:**

We employed a co‐design methodology, bringing together a working group of older adults, primarily those living with dementia, along with their carers/supporters, to adapt the ASCOT toolkit. The adaptation process involved six working group meetings. In between these meetings, three rounds of cognitive testing (Meadows, 2021) with 25 participants who had difficulties completing traditional questionnaires also took place, with findings brought back to the working group after each round, so they could further refine the toolkit in light of cognitive testing results.

**Results:**

The final adapted version of the ASCOT toolkit differs significantly from the original. The cognitive testing results demonstrate a considerable reduction in challenges experienced by participants between testing rounds, indicating a more accessible and user‐friendly tool.

**Implications:**

The findings from this project demonstrate that co‐designing outcome measures with older people, particularly those living with dementia, is both feasible and impactful. This work offers a replicable model for creating inclusive, accessible tools that amplify the voices of service users.

**Patient and Public Involvement:**

8 older people, including those living with dementia, have co‐designed the new version of the tool over 6 meetings. Further meetings took place to jointly design dissemination materials. Working group members have co‐presented project findings at conferences and events, and two members have co‐authored this article. PPI were involved from the funding acquisition stage of this project. Two PPI members who were not part of the working group were part of the project steering group.

An estimated 2.2 million older people in England require support with at least one activity of daily living, with needs met variously by family, friends, or social care services [1]. Over half a million older people rely on publicly funded social care [2], while a significant proportion self‐fund their care, including 33.2% in community settings and 48.9% in care homes [3, 4]. Social care supports older adults in their own homes, residential settings, and community‐based services, providing help with everyday tasks such as personal care and mobility [5, 6]. The overarching goal is to promote wellbeing and enable older people to live fulfilling lives (Age UK, 2023), making it essential to assess whether this care meaningfully enhances quality of life (QoL) [7].

The Adult Social Care Outcomes Toolkit (ASCOT; www.pssru.ac.uk/ascot) was developed to capture the impact of social care on individuals and is used widely to measure social care‐related quality of life (SCRQoL [8, 9];). It measures the aspects of quality of life most impacted by social care and are important to people accessing services, namely: control over daily life, personal cleanliness and comfort, a clean and comfortable home, food and drink, personal safety, social participation, occupation (doing things one values and enjoys), and dignity. A suite of ASCOT tools has been developed for different groups across a range of social care contexts [9]. There are tools aimed at capturing SCRQoL not only of people supported by services [10], but also their carers [11] and paid care staff [12].

The versions include self‐complete questionnaires [8], interview tools (ibid.), structured observation schedules [13] and ways to integrate ASCOT in care planning [14], as well as proxy‐report tools [14, 15] – used where someone who knows the person well (e.g. a family member or a care worker) reports on their behalf, for instance in cases of advanced dementia. ASCOT includes tools tailored for use in both community‐based care (such as domiciliary care) and residential settings (such as care or nursing homes). The toolkit is used in 10 countries besides the UK, with a range of translations and adaptations available, including a version in Braile [9].

Importantly, not all people who find standard questionnaires difficult to complete require a proxy measure (i.e. someone reporting their SCRQoL on their behalf). Self‐completion may still be possible, as long as the barriers associated with standard questionnaires are addressed by prioritising a user‐friendly, accessible design. In this context, ensuring that groups often excluded from research are able to express their SCRQoL is not only crucial for adequately capturing service quality, but also essential to realising their citizenship and social rights [16, 17]. Who is and isn't enabled to self‐report their social care‐related quality of life forms part of a broader conversation about inclusion and participatory rights [18, 19].

With this in mind, an ‘easy‐read’ version of ASCOT was previously developed for people with intellectual disabilities and autism [20]. This easy‐read version featured new images illustrating each ASCOT domain, a consistent layout across all questionnaire items, and modifications to the response scale presentation along with accompanying descriptive statements and visual representations for additional support. Cognitive interviews, a structured “think‐aloud” method using interviewer probes to examine how respondents interpret and answer questionnaire items [21], indicated that this version worked well for the majority of respondents, demonstrating good accessibility and usability among people with intellectual disabilities and/or autism [22].

People with intellectual disabilities are not the only group who may find standard questionnaires difficult to complete. Some older people may also experience challenges due to the cognitive effort required by complex survey questions and could benefit from a more accessible assessment tool [23]. This should not be taken to mean that all older people face such difficulties or to reinforce ageist assumptions; age alone does not predict performance on scale‐based questionnaires, regardless of question length or complexity [24]. Instead, the ability to self‐complete questionnaires depends on cognitive and communication capacities [23].

While estimates vary, around 10%–20% of people aged 65 and over may have Mild Cognitive Impairment (MCI), which is characterised by a deterioration in memory, attention and cognitive function [25]. It is also estimated that 60% of people receiving home/domiciliary care services [26] and 70% of people residing in care or nursing homes live with a form of dementia [27]. It is this subgroup of older people that may find standard questionnaires challenging [28]. Proxy‐report tools may be necessary for people living with severe dementia, whose communication may be substantially affected by the illness progression, but should only be used where self‐report is not possible [10, 14, 29, 30]. However, for those living with MCI, mild to moderate dementia, or other age‐related communication difficulties, more accessible self‐completion questionnaires are needed to ensure that their perspectives are captured.

Provider organisations have highlighted the need for an easy‐read ASCOT tool for older people who struggle with the standard version. The ASCOT team had also received enquiries about whether the existing easy‐read version, developed for people with intellectual disabilities and/or autism, could be used with older people. However, this ASCOT‐ER version included mostly people under the age of 65, with elements such as illustrations developed specifically with this group in mind. It was not deemed suitable for an older adult population.

While some broad guidelines on ‘universal accessible approach' to questionnaire design exist (e.g [31]), these have been developed with learning disabilities in mind. What constitutes accessible design can vary widely depending on the specific challenges or conditions a person experiences, as well as their individual needs [32]. For example, someone with visual dyslexia may require coloured prints or overlays, while phonological dyslexia may benefit from specific fonts and active rather than passive language style [33]. Designing separate tools for each condition or challenge would, of course, not be feasible. However, different design principles do apply depending on whether the communication difficulty is life‐long (e.g., those associated with intellectual disabilities) or acquired later in life (e.g., due to MCI or dementia). This highlights the need for a tailored approach focused specifically on older people who may struggle to complete standard questionnaires due to MCI, dementia or other challenges developed later in life.

Dementia advocacy organisations offer guidance on making information more accessible (e.g [34, 35]). However, designing accessible survey tools involves unique challenges that go beyond the considerations involved in producing general information materials [36]. Patient and public involvement – and more specifically co‐design ‐ of accessible survey tools can be particularly beneficial where general guidelines do not suffice (e.g [37]). While such involvement is increasing in self‐report tool/scale development [20, 37, 38, 39], co‐design with people with lived experience remains relatively rare.

Our aim was not only to make self‐report tools for assessing SCRQoL more accessible to older people who may find them difficult to complete, but also to do so by involving people with lived experience directly in the design process. We believed that co‐design is the most inclusive, ethical and effective way to develop accessible tools [37]. Co‐design in health and social care is conceptualised in a variety of ways [40], but is broadly defined as a participatory design methodology involving ‘end‐users’ in research or service design [41]. The process extends beyond consultation, which typically leaves researchers or service providers responsible for ‘translating' consultation findings. Rather, it is characterised by collaborative working across multiple stages, with participants actively involved in clearly defined tasks, such as ideation and iterative prototyping of interventions, care pathways, or research tools [42].

The aim of this project was to co‐design an easy‐read self‐report ASCOT questionnaire for and in collaboration with older people, including those living with dementia. This paper outlines the co‐design process, who was involved, the outcomes, and reflections from the co‐design Working Group.

## 1 The Working Group

The co‐design Working Group (WG) was established as part of a research project aiming to adapt and assess the content validity of the [redacted for anonymity] Easy‐Read self‐report version for older people accessing social care. While the overall project constituted a research activity (see [redacted for anonymity] for details), the activities of the WG were conducted as part of Patient and Public Involvement (PPI). Ethical approval for the entire study was granted by [redacted for anonymity].

The co‐design Working Group (WG) of people with lived experience was assembled by co‐applicant EF, a clinical psychologist, through local Dementia Engagement and Empowerment Project (DEEP) groups and family carer support networks. Some WG members already knew each other through these networks. Many of these individuals had already taken part in a lived experience group (*N* = 6) who helped shape the funding application for this project and helped confirm from a PPI perspective that an Easy‐Read tool specific for older people was required. Importantly, when asked if any group members would have liked to become study co‐applicants, none felt able to take on this degree responsibility, citing in particular the possible progression of their own dementia or that of the family member they were supporting, which made a 2 year commitment difficult. Instead, the group elected to stay as a group, sharing responsibilities across a greater number of people.

Once the project was funded, nine people expressed an interest in attending co‐design workshops, with eight becoming regular members, missing one or no meetings. The ninth person attended the second meeting but did not return, citing poor health. References to the WG relate to the eight regular members. All working group members were aged over 65, reflecting the toolkit's intended user population. Half were people living with dementia; two were current carers, and one was a bereaved carer. One member used a handheld optical magnifier when reading documents. There was considerable variation in prior research experience among WG members. One person had extensive and recent experience advising and participating in research; another had past professional research experience. A spousal couple had taken part in a clinical dementia trial. Other group members did not have prior experience of research and none of the WG members had been part of co‐design activities specifically. As WG members were part of the co‐production team, not research participants, we did not collect demographic data.

Aside from age being used as eligibility criteria for the study, we did not collect WG member demographic data, such as gender or ethnicity. Neither gender nor ethnic background can ‐ or should ‐ be inferred from observation as they are a self‐identified constructs. The sample nevertheless appeared heterogeneous in terms of gender but homogeneous in ethnic background, reflecting the majority ethnic group of the study setting.

Eight meetings took place between May 2022 and February 2024. The first six focused on development and refinement of the easy‐read tool. The final two meetings involved further refinements, co‐designing an accessible project summary for the public [43], and filming video testimonials with six members who volunteered [44].

Meetings were held at a familiar local community centre and lasted up to 3 h, including breaks and time for socialising. Following consultation with EF and the WG, meetings began at 10am to reduce cognitive fatigue (commonly experienced later in the day by people with dementia) and allow preparation time. Between three and five project team members attended each WG meeting. JC led and chaired meetings, with contributions from other team members. All team members were experienced in working with people living with dementia, and most had expertise in co‐design. WG members were paid at NIHR INVOLVE rates and travel was reimbursed.

## 2 Co‐Design Process

We employed an agile, iterative co‐design process, previously shown to work well with people living with dementia [45]. The WG played an integral role in the iterative development and refinement of the revised ASCOT‐ER measure. In meetings 1–3 WG members reviewed the original ASCOT Easy‐Read tool developed for people with intellectual disabilities and/or autism [20] alongside the standard ASCOT‐SCT4 questionnaire [8]. This input led to the initial draft of the new, adapted tool, which was then applied in qualitative interviews using cognitive interview methodology [21]. Specifically, interview participants were asked to complete the questionnaire and ‘think aloud’ whilst doing so. The interviewer then probed, as needed, to explore the participant's understanding of the question and its layout, any recall required or weighing up of response options and how they would indicate and record their response [46]. Between meetings, the research team implemented suggested revisions by the group, sometimes offering alternative design options where views among WG members differed.

Further WG meetings (4–6) took place after each round of cognitive interviews. During these, the research team presented findings from the interviews (e.g., identifying items participants found confusing or hard to interpret) and sought WG guidance on how to revise the tool. When changes involved layout or sequencing of elements, multiple printed options were offered and WG members invited to compare and explain their preferences.

**Figure 1:**
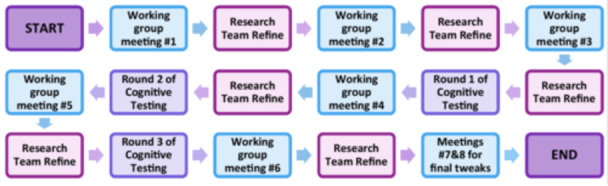
Process of ASCOT‐ER (Older People) tool design; figure co‐designed with the working group.

To illustrate the sequence of WG meetings, cognitive testing rounds, and tool refinements, the WG developed a visual representation of the process (Figure 1). The content and design of the graph were discussed in WG Meeting 7, with Meeting 8 used to review draft content, select preferred layout, and suggest further refinements e.g. adding ‘start’ and ‘end’ boxes and emboldening boxes.

## 3 Results

### 3.1 Co‐Design Outcomes

During the WG meetings, members made numerous suggestions, ranging from general (e.g., text and image layout on the page) to highly specific ones (e.g., wording of sentences). Lengthy discussions and divergent opinions were common, but there were no acrimonious conflicts at any of the meetings. Meeting facilitators (typically JC) allowed adequate time for each discussion topic and kept interruptions or steering from the research team to a minimum. When the facilitator or other research team members spoke, it was primarily to clarify points, reiterate the rationale behind the activity, and ensure the changes proposed by the WG members were properly captured by the researchers.

Boxes 1 and 2 below provide a ‘snapshot’ example of one such discussion. It relates to a proposed addition of a clarifying sentence to the ‘dignity’ item of the tool. Round 2 of the Cognitive Testing interviews revealed that some participants did not distinguish between paid and unpaid types of support (the latter provided by family members or neighbours) when answering the question about paid support. This is understandable, given that most people receiving paid social care support are also supported by family members, friends, and/or neighbours [1]. However, the ASCOT tool is specifically concerned with quality of life related to paid social care provision. Therefore, a further clarification of the question was necessary.

Box 1 **Changes to the ‘Dignity’ Question during Working Group meeting number 5.**

**Version used during cognitive testing:**

How do you feel about the way your paid support treat you?

**Proposed amendments discussed in WG meeting no5:**

*Version 1:*

How do you feel about the way your paid support treat you? By paid support we mean anyone that is paid by you, or anyone else (including the council), to support you.

*Version 2:*

How do you feel about the way your paid support treat you? By paid support we mean anyone that is paid to support you. This could be by you, or anyone else (including the council).

**Version agreed upon by the WG at meeting 5** (further changes were made at subsequent meetings):

How do you feel about the way your paid support treat you? By paid support, we mean any person, groups, or service that is paid for by you, your family, or anyone else, including the council, to support you.

Box 2 **Quotes from the discussion on *‘Dignity’ Question during Working Group meeting number 5*.**

**Table:** 

WG member 1	*“I think version 1 is the best”*
WG member 2	*“I like version 1”*
WG member 3	*“I like version 2, but is it necessary to put in brackets ‘including the council”? It could just read: ‘or anyone else, including the council’”*
WG member 4	*“Jogs your memory though, doesn't it?”*
WG member 3	*“Then [without the brackets] it just runs on”*
WG member 2	*“Version 2 reads a bit clumsy”*
WG member 5	*“I think version 1 is really to the point. Maybe I'm getting a bit tired, but I found version 2 confusing”*
WG member 1	*“It's where you put ‘to support you’”*
WG member 6	*“I thought that. Version 1 splits them up better… it leaves you with the final statement of support, whereas in version 2 ‘to support’ comes earlier”*
WG member 5	*“I've read version 2 several times… possibly if I've read it at the beginning of the sessions… but at the moment, and let's face it, people that are doing it are gonna be like me [i.e. living with dementia] and they might get tired. I prefer version 1, I find that I understand what it says”*
WG member 1	*“[Version 1] It is punctuated by the comma. I think version 2… what you are trying to do there is to qualify what you mean: ‘this could be you or anyone else’”*
WG member 6	*“It's the order that's important. I think version 1 is better because the two key words, well, the key word is ‘support’ and version 1 begins with talking about support, and it ends talking about support. Version 2 ends talking about the council. So you are left with this thought about the council… which is important, but of secondary importance”*
WG member 2	*“I prefer version 1. I think version 2 reads clumsy. I think if you were writing version 2, after ‘support you’, if you were writing it, instead of a full stop you'd put a dash to qualify”*
JC (facilitator)	*“Ok… so I feel like we are sticking with version 1. Are we thinking about removing the brackets then and instead using a comma?”*
WG member 1	*“You're just taking out the words ‘this could be you’… and the brackets”*
JC (facilitator)	[Reads out the full revised revision. The group agrees with nods and audible ‘yeses’]
WG member 5	*“I would not even notice the grammar and the comma. I can get the sense of what it's saying. That I can take in and understand, even when I'm getting tired”*
AMT (research team member)	*“I am really fascinated by this. I can assure you, if we published that version 1, so many people would criticise and say to us ‘you have not used accessible format, you have not used short sentences’. So it is really useful for us to have this, because we have to be really clear that this has come from the group, that we compared two versions”*
EF (research team member)	*“Yes, because those of you living with dementia were much more ‘definitely need version 1, and those of us who don't go ‘oh, version 2 might not be too bad’* [all laugh]
WG member 5	*“I kept reading that and it just did not make any sense to me*.

On this occasion, the research team offered the WG two alternative versions as a starting point. This approach meant that WG members did not need to figure out how to proceed from scratch or choose between a single version to accept or amend. Having two versions already presented as part of the entire item (i.e. including images, descriptions, questions, and response items) allowed WG members to consider the proposed changes in context and express a preference for one version over the other. However, unlike consensus‐based methodologies often used in co‐design (e.g. [47]), this process encouraged and actively allowed further changes to be made or for both alternatives to be rejected.

As can be seen in Box 2, there was an initial divergence of opinion, with some WG members preferring version 1 and others favouring version 2. In this case, as in many others, the group decision was not merely a matter of majority preference. In cases of differing opinions, it was typical for WG members to support the option preferred by those living with dementia.

**Figure 2:**
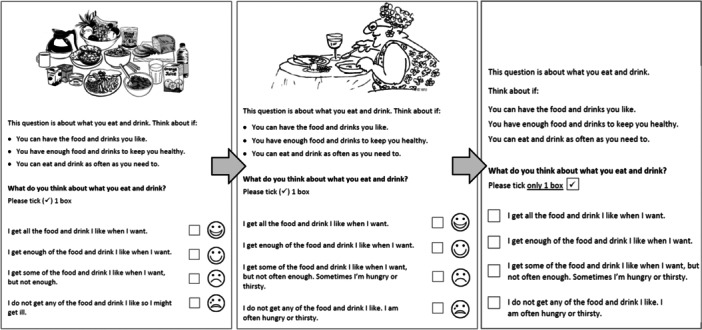
Changes to the ‘Food and Drink’ Item (left: original easy‐read version for people with intellectual disability; centre: version v1.3 used for round 1 of cognitive testing; right: final version v1.7).

Extensive changes were made to adapt ASCOT‐ER (OP) tool. Images used in the original easy‐read version [20] were replaced with illustrations depicting older people, adapted from versions used in Germany and Austria [48]. However, cognitive testing [21] showed that many older people found the images of no additional value or distracting/“silly” [46]. These findings were shared with the Working Group, who were divided on their added value. As a result of this WG feedback, the last round of cognitive testing presented participants with two versions ‐ one with illustrations and one without ‐ and asked them to choose the preferred version. Most participants chose the non‐illustrated version. This was then shared with the WG for further discussion, at which point the group decided to use the version without illustrations. Other changes included the addition and/or reordering of prompts for questions (as exemplified in Box 1), changes to the wording of the response options, removing ‘smiley faces’ and qualifiers (e.g. ‘It is okay’), and the relocation of the tick‐boxes to the left. Figure 2 showcases the evolution of the ‘food and drink’ item (for a comprehensive list of changes made to each toolkit item, see [46]).

The cognitive testing process demonstrated that with each iteration, the tool became more relevant, more understandable, and easier to respond to than ever before for this group of people. The number of issues participants experienced reduced significantly, particularly for those participants experiencing a greater level of cognitive impairment, from round 1 to round 3 of Cognitive Testing (to read more about participant characteristics and changes in the frequency of difficulties with filling in the tool, see [46]).

Once the toolkit design was finalised, some WG members remained involved in dissemination. The project summaries for the public [43] and the social care sector [49], as well as video testimonials [44, 50], were developed with WG member involvement. One working group member gave a presentation about the project at an online webinar together with JC. Another WG member attended two in‐person academic conferences and presented alongside RM. Conference attendance was enabled by the ability to not only fund conference registration, travel, and accommodation for a working group member living with dementia, but also for a volunteer supporter.

### 3.2 Group Member Experiences

Working group members were invited to record evaluative testimonials, reflecting on their experiences of taking part. To facilitate this process, they were asked three questions: (1) What has being part of this research study been like? (2) Are there any aspects you particularly enjoyed or did not like? and (3) Would you recommend getting involved in research to others? Six WG members took part.

While this was not explicitly asked in the questions outlined above, many WG members reflected on why they took part in the co‐design of the new tool. The desire to contribute and make a difference, as well as the wish to help others in similar circumstances (even without any direct benefit for themselves and even if any resulting changes will take a long time), were frequently mentioned.

I think the fact that I thought that I could bring something to the group, and so that's my that was my original intention.

(WG4)

Since [my husband] was diagnosed, we've been keen to be involved in research, because even if it's something that doesn't help him, I think research is quite essential to […] find ways of solving things.

(WG2)

Practical aspects of participation were also important. Those few WG members who had experience of research already, but usually online, valued this being a local, in‐person project with an easy‐to‐get‐to venue. People living with dementia on the WG also appreciated being able to take part in the project together with their spouse (as opposed to co‐design work only involving those living with dementia). Some mentioned trusted relationships with EF, as encouraging participation and alleviating fears.

We asked WG members what participation was like and what they enjoyed/did not enjoy. A sense of purpose and meaning from the ability to contribute was mentioned the most frequently:

It saved my life. It made me look… I have Alzheimer's, and I thought it was the end of my world, but I've just realised it's not. This is just the beginning of a different world, and coming to this [the Working Group] was the first step of that, and it's been really life changing. And if I can be involved in things like this, gives me a reason to keep going.

(WG5)

Members valued the collaborative process of the meetings and positive group dynamics. Some also mentioned the importance of gaining new knowledge and perspectives, both from other WG members and from researchers.

The group itself has been quite diverse, and I found that really helpful, and I think probably the researchers did as well. I know that we've been throwing ideas at you, but actually you've come back as professionals and stopped us in our tracks and made us think […] and we've let the researchers have an input as well. That's been brilliant.

(WG1)

WG members expressed a particular interest in the iterative process of the project and the way the cognitive testing rounds were integrated with the co‐design work. WG members identified some initial difficulty with balancing different opinions. However, appreciating the collaborative process of the co‐design helped them to enjoy and value the process regardless. The loss of illustrative images on the tool was one example given for this process:

I personally felt a great deal of attachment to the illustrations, but it was good for me to know that they weren't going to be used […] I was reminded of the process and the views of [cognitive testing participants] and how, in fact, the piece of work was far more important than my own personal thoughts on what I've contributed.

(WG6)

WG members who took part in the evaluation also unanimously endorsed the involvement of others in research. Some spoke of talking about their experiences with this project with their friends and relatives and encouraging them to participate in research

I'm really glad to be able to come and talk to my friends […] and if it means that people have a better understanding of how decisions are made and whether they might want to also participate in research projects like I have been doing, then I think that must be a really good thing.

(WG3)

The enjoyment of the co‐design process itself and the empowering experience of feeling treated as an equal by the research team and others in the group were the main reasons for remaining involved in the project:

I've had Alzheimer's for quite some time, and people tend to look at you as if you're stupid, but to be treated as an equal and to have your opinions listened to and sometimes taken note of is really good for you, because […] this makes you think that you're not as silly as you think you are, that you can still contribute.

(WG5)

The reflections above were captured at WG meetings 7 and 8, as the project was coming to an end. As covered in the previous section, some WG members took part in further dissemination, namely webinar and conference presentations. In preparing this paper, our study has also benefited from retrospective reflections of two working group members, written a year after the formal end of the project.

Box 3 offers reflections from two working group members, written a year after the last WG meeting had taken place.

Box 3 **Reflections from Keith and Rosemary Oliver.**

We always looked forward to these meetings as we knew most of the participants really well and a genuine spirit of trust, respect, consideration and tolerance was built during the course of the project. This really enabled everyone to be open and honest in their thoughtful contributions, and whilst there were occasions when a participant's views were in a minority, including sometimes our view differing from others, for example around the use of pictures and emojis, we were comfortable with hearing different views and then decisions taken resting upon the majority.

The issues the toolkit covered are very relevant to us given our age, one of us living with dementia and the other with physical disabilities through ageing. So we did invest heavily in the project and took it very seriously. There was 100% commitment from us both at all of the meetings over the 21 month period of the project, and we felt this applied throughout the lived experience group to ensure that the toolkit was fit for purpose, and would support other people living with dementia and family care givers needing to access services to retain their independence.

## 4 Discussion

This project has shown that co‐designing questionnaire‐based tools to assess social care related quality of life is both feasible and highly effective; and that it can be applied in a different, more extensive and iterative approach than previously used (e.g [20]). The process supports researchers during development, produces a more accessible and user‐friendly end product, and brings meaningful benefits to the experts by experience who are involved.

While the inclusion of people with dementia in Patient and Public Involvement and Engagement (PPIE [51]) is growing, participation remains uneven across disciplines, research types, and stages of the PPIE process [52]. Co‐design of assessment tools is one such area where involvement of experts by experience, let alone those living with dementia, is on the rise (e.g [37]) but remains relatively uncommon. This gap speaks to wider issues of citizenship in research [53, 54]. Combining researcher expertise with lived experience enhances both epistemic justice and a form of inclusion [55, 56], with our work showing that such an approach is not only just and ethical, but doable. Outcome measures can and should be co‐developed with the populations they are intended to serve, as has been successfully demonstrated before [20, 37, 38, 39]. A diagnosis of dementia does not ‐ and should not ‐ preclude meaningful involvement.

While we show that meaningful expert‐by‐experience involvement in co‐designing assessment tools is achievable, we do not suggest it is easy or possible without due care and consideration. Reflections from our WG members highlight the importance of both practical and interpersonal elements in making this work. In‐person, local meetings were particularly valued, especially in light of the relatively recent COVID‐19 restrictions on gatherings. For many, familiarity and a degree of trust in at least one team member, as well as the option to participate alongside a spouse or supporter, were key motivations for joining the WG. Meanwhile, the iterative nature of the meetings (rather than a one‐off consultation), combined with time and space for informal interaction, helped build trust among WG members and between the WG and researchers. This created a space where challenge and disagreement felt safe and constructive. A collaborative approach, and the shared sense of making a difference to others (even without immediate benefits), played an important role in sustaining engagement. Buy‐in was also strengthened by involving some WG members from the funding application stage through to dissemination.

The importance of trust, relationship‐building, an iterative approach across project stages, and co‐ownership of decision‐making are echoed throughout the literature on PPIE. In this sense, our observations are not novel. Nonetheless, our work adds to the PPIE discourse in a number of ways. Firstly, we show that similar principles can be successfully applied to the development of patient reported outcome tools, specifically in the form of integrating and iterating co‐design and cognitive testing. Secondly, we show that mixed group membership, where only some members had a diagnosis of dementia, did not impede the process or the outcomes. In cases of diverging opinions, the group often prioritised the perspectives of those living with dementia, demonstrating both the feasibility and the value of such ‘mixed‐group’ involvement. Lastly, we show that a working group can be a suitable alternative to the more typical involvement of a smaller number of lay co‐applicants in the project team, as it reduces individual burden and allows responsibilities to be shared across lived experience representatives.

As well as contributions to the literature on PPIE, our paper also offers insights into accessible design. The ASCOT easy‐read version for older people supports the team's initial view that the existing easy‐read tool, developed for people with intellectual disabilities and/or autism, was not a suitable fit for older adults who may struggle with standard surveys due to dementia, mild cognitive impairment, or other age‐related difficulties. This new version differs significantly from both the easy‐read tool for people with intellectual disabilities and/or autism (ASCOT‐ER [20]) and the standard self‐complete survey (ASCOT‐SCT4 [8]). The former raises questions about how universal the ‘universal design principles’ really are [20]. Our findings suggest that what makes survey content accessible to people living with dementia or mild cognitive impairment is distinct, likely because these challenges develop later in life, when individuals may draw on a wider range of compensatory strategies. For instance, long sentences, typically discouraged under universal design principles, were preferred by our working group and found helpful by those in cognitive testing rounds, as they offered more explanation. Rather than causing confusion, additional information often provided clarity. Conversely, non‐text elements, especially illustrative images, which are often informative for people with intellectual disabilities, had little benefit or were distracting for our participants.

This does not mean that the revised tool will suit all older adults equally well. Many older people are intellectually disabled, autistic, or have specific learning difficulties such as dyslexia [57, 58, 59]; identifying the most suitable easy‐read version/format for these sub‐populations should considered in future research applying the measures in research with diverse older adults. Nonetheless, the need to distinguish between design considerations for lifelong versus later‐life cognitive disabilities is clear and should be recognised. This further underscores the need for co‐design to ascertain what is accessible, for whom and in what way. While the cognitive interview process alone [21] would have allowed us to iteratively incorporate feedback from people with lived experience, how to incorporate feedback would have been left to the researcher; blending cognitive interviewing with co‐design in the current study has instead allowed us to share decision‐making and move toward a more democratic distribution of power in research.

While this study offers a number of contributions to research, as discussed above, the limitations of our work should also be acknowledged. While social care supports individuals at all stages of dementia and older adults without dementia, the easy‐read tool is specifically intended for people in later life who receive social care and struggle to complete standard surveys. In relation to this, it is worth noting that WG members had limited personal experience of using social care services. As such, in terms of familiarity with social care, our WG did not fully reflect the tool's target user group. Nonetheless, we know that the WG input made the tool more accessible to this target user group. The cognitive testing process [46] showed substantial and increasing improvements in participants' ability to complete the tool across rounds. The difference in support needs or degree of cognitive challenges between the WG and the cognitive testing participants, therefore, did not nullify the impact that lived experience perspectives had on the tool's design.

It should also be noted that we did not collect demographic characteristics from our Working Group. As such, no inferences can be made about participants' identities based on observation alone. However, given that the study was conducted in a region where certain demographic groups are demographically and institutionally dominant, the Working Group appeared relatively homogeneous with respect to some characteristics, which may have shaped the perspectives represented. Future work should explicitly attend to how structural dimensions of identity shape participation in tool co‐development and its results.

## 5 Conclusion

To conclude, co‐designing person‐reported outcome measures, like ASCOT, is doable and produces improved outcomes. Not only can co‐design be achieved, but it can be done in an extensive, iterative way, that spans across the research cycle from idea generation and funding acquisition, all the way to dissemination and impact – fully applying the patient and public involvement principle of ‘nothing about us without us’ to assessment tool design.

## Author Contributions

**Rasa Mikelyte:** conceptualisation, funding acquisition, investigation, resources, visualisation, writing – original draft. **Keith Oliver:** conceptualisation, investigation, funding acquisition, writing – original draft, visualisation. **Rosemary Oliver:** conceptualisation, funding acquisition, investigation, writing – original draft, visualisation. **James Caiels:** Conceptualisation, data curation, formal analysis, funding acquisition, methodology, project administration, resources, validation, visualisation, writing – review and editing. **Stacey Rand:** conceptualisation, data curation, formal analysis, investigation, methodology, project administration, validation, visualisation, writing – review and editing, funding aquisition. **Elizabeth Field:** conceptualisation, funding acquisition, resources, writing – review and editing. **Lucy Webster:** conceptualisation, data curation, formal analysis, investigation, resources, validation, writing – review and editing. **Ann‐Marie Towers:** conceptualisation, investigation, funding acquisition, writing – review and editing, visualisation, validation, methodology, project administration, resources.

## Ethics Statement

The authors declare that ethical approval for the study granted by the Coventry and Warwick Research Ethics Committee on 14th December 2022 (Reference [17]/WM/0234). The authors declare that consent to publication of findings – including quotations and any personal or identifiable information – was secured prior to publication.

## Conflicts of Interest

The authors declare no conflicts of interest.

## Data Availability

The data that support the findings of this study are available from the corresponding author upon reasonable request and with research ethics board approval.
